# Development of local strontium ranelate delivery systems and long term *in vitro* drug release studies in osteogenic medium

**DOI:** 10.1038/s41598-018-35197-7

**Published:** 2018-11-13

**Authors:** Dagnija Loca, Anastasija Smirnova, Janis Locs, Arita Dubnika, Jana Vecstaudza, Liga Stipniece, Elina Makarova, Maija Dambrova

**Affiliations:** 1 Rudolfs Cimdins Riga Biomaterials Innovations and Development Centre of RTU, Institute of General Chemical Engineering, Faculty of Materials Science and Applied Chemistry, Riga Technical University, Pulka Str 3, Riga, LV-1007 Latvia; 2 Latvian Institute of Organic Synthesis, Laboratory of Pharmaceutical Pharmacology, Aizkraukles Str 21, Riga, LV1006 Latvia; 3 Riga Stradins University, Faculty of Pharmacy, Dzirciema Str 16, Riga, LV1007 Latvia

## Abstract

It has been recognized that the operative stabilization of osteoporotic fractures should be followed up with an appropriate osteoporosis treatment in order to decrease the risk of repeated fractures. Despite the good clinical results of strontium ranelate (SrRan) towards the osteoporosis treatment, high drug doses and long treatment period cause an increased risk of serious side effects. Novel local SrRan/poly(lactic acid) (SrRan/PLA) delivery systems containing from 3.57 ± 0.28 wt% to 24.39 ± 0.91 wt% of active substance were developed. In order to resemble the naturally occurring processes, osteogenic media (OM) was used as a release medium for long term (121 days) *in vitro* drug release studies and UV/VIS method for the determination of SrRan content in OM was developed and validated. Biomimetic calcium phosphate precipitates were found on the surface and in the pores of prepared delivery system after microcapsule exposure to OM for 121 days as well as SrRan particles, indicating that the release of the drug have not been completed within 121 days. *In vitro* cell viability evaluation approved no cytotoxic effects of microcapsule suspensions and extracts.

## Introduction

Stabilization and treatment of osteoporotic bone fractures still remains a big challenge. Osteoporosis is usually associated with low bone mineral density, resulting in reduced bone strengths and increased risk of bone fractures^1^. It has been recognized that the simple treatment of the acute fracture is insufficient and in order to decrease the risk of repeated fractures, it should be followed up with the appropriate osteoporosis treatment^2^. Current approaches for the treatment of osteoporosis include wide range of methods, for instance, non-pharmacologic approach by resistance and weight-bearing exercises as well as vitamin D and calcium treatment, but more commonly pharmacological therapies applying drugs with antiresorptive or anabolic effect are used (Table 1).

**Table 1:** Pharmacological therapies for the treatment of osteoporosis.

Pharmacologic therapy	Active molecule	Results from clinical trials	Reference
Antiresorptive	Raloxifene	Inhibits bone resorption, but increases thromboembolic events	^32, 33^
	Alendronate	Reduces the frequency of morphometric and clinical vertebral fractures	^32, 33^
	Risedronate	Reduces risk of vertebral and nonvertebral fractures	^32, 33^
	Ibandronate	Reduces the rate of vertebral fractures, increases total bone mineral density	^32, 33^
	Zoledronic acid	Reduces vertebral and nonvertebral fractures, can cause an acute-phase reaction, thus coadministrated with acetaminophen	^32, 33^
	Denosumab	Inhibits osteoclast activation, reduces vertebral, nonvertebral and hip fractures	^32, 33^
Anabolic	Teriparatide (PTH (1–84) and PTH (1–34))	Reduces vertebral and nonvertebral fractures, increases bone mineral density, can be used in patients with severe osteoporosis for rapid bone growth	^32– 34^
Antiresorptive as well as anabolic properties	Strontium ranelate (SrRan)	Increases bone mineral density, improves intrinsic bone tissue quality and microstructure	^33^

Conventional methods of supplying a patient with pharmacologic substances usually are through injections, oral ingestion or infusions. These methods often are poorly selective, thus the side-effects and damage can occur to the healthy tissues and organs. In addition, high drug doses can be required to achieve the desired effect^3^. Although most of the drugs summarized in Table 1 have demonstrated the antiosteoporotic effect in clinics and are recognized to be effective for the osteoporosis treatment, not all of them promote both the bone fracture healing and further bone remodelling. Despite promising results of pharmacological therapies with bisphosphonates, the side effects and complications are present, such as atypical femur fractures and osteonecrosis of the jaw^4^. Moreover, essential issue is oral absorption, which ranges from 0.7% for alendronate and risedronate to 6% for etidronate and tiludronate^5^. Thus, new treatment directions, optimal doses, possible toxicity and effective delivery strategies based on the use of implantable delivery tools able to release the active substance in a controlled way are under development^6^.

SrRan is thought to have both antiresorptive and anabolic properties and therefore increases and stabilizes bone mineral density. Still, the mechanism of SrRan action is under investigation and mainly could be related with increased strontium content in the bone^7,8^. Strontium positive effect on the bone remodelling has been proved in the previous studies through reducing osteoclast differentiation, activity and bone resorption *in vitro* as well as increasing osteoblast differentiation^9–11^. SrRan consist of two strontium atoms that are stabilized with ranelic acid and despite its good clinical results towards osteoporosis treatment there is an increased risk for side effects, including venous thrombosis, diarrhoea, nausea, headache, cutaneous hypersensitivity^12–14^. Moreover, an increased risk for myocardial infarction have been detected in randomised clinical trials^15^. Nevertheless, SrRan treatment has to be ensured for a long term, at least for 3 years, with a *per oral* dose of 2 g/day^8^. In order to decrease the daily dose of drug as well as to avoid the pronounced side effects, development of long term local SrRan delivery system would be of clinical importance.

There are many researches, showing development of local bisphosphonate delivery systems. For instance, Balas *et al*. used alendronate as a model drug and prepared local delivery system by loading it in siliceous ordered mesoporous materials. For such system the drug release can be ensured only up to 12 days^16^. Other alendronate delivery systems have been prepared using such microencapsulation techniques as water-in-oil-in-water (w_1_/o/w_2_), water-in-oil-in-oil (w/o_1_/o_2_), solid-in-oil-in-oil (s/o_1_/o_2_) and solid-in-water-in-oil-in-water (s/w_1_/o/w_2_), applying poly(lactic-co-glycolic acid) (PLGA) as a carrier^17,18^. These systems showed better drug loading efficiency (~73%) as well as (s/w_1_/o/w_2_) ensured *in vitro* drug release up to one month with initial burst release of 9–20%^17^. Miladi *et al*. has reviewed 17 studies on development of microencapsulated antiosteoporotic drug delivery systems and 18 studies using other delivery system preparation methods.

Recently, Mao *et al*. published research, where SrRan was encapsulated in poly(lactic-co-glycolic acid) microspheres in size of about 150 μm, reaching drug loading of Sr 0.92 ± 0.14 atomic concentration %^19^. Although drug release for 22 days was presented, it might be not long enough, as efficiency of SrRan has been demonstrated in long term treatment^11^. Nair *et al*. attempted to prepare local SrRan delivery system based on polycaprolactone-laponite composite scaffold. Obtained scaffold showed promising results, with initial burst release of 10–20%, but the cumulative release was studied in water^20^, revealing a common problem for antiosteoporotic drug detection methods. Due to low solubility of bisphosphonates and SrRan in biological fluids^21^, detection methods are usually developed in water^22,23^. Bisphosphonate detection in biological fluids usually includes advanced sample preparation, methods and instruments, such as, liquid or gas chromatography, capillary electrophoresis, mass spectrometry and inductively coupled plasma analytical methods^24^. For cumulative release studies *in vitro*, method of active substance detection in osteogenic medium is highly necessary.

In the current research SrRan was encapsulated in poly(lactic acid) (PLA) matrix applying s/o/w and s/w_1_/o/w_1_ techniques. Moreover, simple, fast and reliable UV/VIS analytical method for the determination of SrRan content in osteogenic medium (OM) has been developed, validated and applied for the evaluation of drug release kinetics for more than 120 days in OM.

## Materials and Methods

### Materials

PLA (Biomer L9000) with molecular weight of 200–300 kDa and polyvinyl alcohol (PVA) with molecular weight of 25 kDa (98 mol % hydrolyzed) were purchased from Polysciences (Warrington, FL). SrRan (LOT Nr.: 140401SR) was purchased from Zhishang Industry Co., Ltd (Shandong province, China). Dichloromethane (≥99.8%), NaHCO_3_ (≥99.7%), β-glicerophosphate (≥99.0%) and L-Ascorbic Acid -2-phosphate (≥95%) was applied from Sigma-Aldrich (St. Louis, MO). Dulbecco’s Modified Eagle Medium (DMEM), FBS Superior FCS Serum, Penicillin/Streptomycin (Pen Strep, 10000 U/ml /10000 µg/ml) and dexamethasone were purchased from Biochrom (Merck Millipore, Germany).

### Microencapsulation of SrRan in PLA matrix using solid/in oil/in water (s/o/w) technique

SrRan loaded PLA microcapsules were prepared using slightly modified microencapsulation method described in our previous study^25^. Briefly, PLA (1 g) was dissolved in 10 ml of dichloromethane. PVA (4 g) was dissolved in 100 ml of water. SrRan (100 mg, 500 mg or 1000 mg) was properly homogenized with 7% PLA solution in dichloromethane (s/o) at 7000 rpm and added to the 100 ml of 4% aqueous PVA solution. Prepared solid-in-oil-in-water (s/o/w) system was homogenized at 7000 rpm and after emulsification, the organic solvent was extracted in 2 L of water. Microcapsules formed were separated by centrifugation for 1 min at 1000 rpm and dried at 40 °C for 24 h.

### Microencapsulation of SrRan in PLA matrix using solid/in water/in oil/in water (s/w_1_/o/w_2_) technique

PLA (1 g) was dissolved in 10 ml of dichloromethane. PVA (4 g) was dissolved in 100 ml of water. Ultrasound homogenization (ultrasonic processor UP200St equipped with sonotrode S26d2, Hielscher, Germany) was used for the preparation of s/w_1_ phase, mixing 100 mg (SrRan 4 wt%), 500 mg (SrRan 16 wt%) or 1000 mg (SrRan 24 wt%) of SrRan with 1 ml of water. Obtained aqueous suspension of SrRan was homogenized for 30 s at 7000 rpm with 7% PLA solution in dichloromethane (s/w_1_/o), added to the 100 ml of 4% aqueous PVA solution and formed s/w_1_/o/w_2_ double emulsion was homogenization for 60 s at 7000 rpm. After emulsification, the organic solvent was extracted in 2 L of water for 60 min. Then the microcapsules formed were separated by centrifugation for 1 min at 1000 rpm and dried at 40 °C for 24 h.

### Characterization of microcapsules

Microanalysis (apparatus – Vario MACRO CHNS, Hanau, Germany) was used to determine the nitrogen content in samples and the total drug load (DL) in microparticles was calculated according to equation 1:

1 $${\rm{DL}}\,( \% )={{\rm{N}}}_{{\rm{el}}}/{{\rm{N}}}_{{\rm{tot}}}\cdot 100,$$

where N_el_ is nitrogen content found using microanalysis and N_tot_ is the calculated nitrogen content in SrRan.

The average microcapsule size and particle size distribution was determined using laser particle size analyzer (ANALYSETTE 22, measuring range from 0.01–1000 μm, laser wavelength 650 nm). Each sample was measured in triplicate.

Surface morphology and inner structure of microcapsules were examined using scanning electron microscopy (SEM, Tescan Mira\LMU, Czech Republic) at acceleration voltage of 3–7 kV. Each sample was sputter coated with 15 nm thin gold layer prior to imaging.

The phase composition was analyzed using X-ray powder diffractometry (XRD, PANalytical X’Pert PRO,Westborough, MA). XRD patterns were recorded using Ni-filter and Cu Kα radiation at 40 kV and 30 mA, 2θ range of 5–60°.

Fourier transform infrared (FT-IR, Varian 800, Scimitar Series, USA) spectra were recorded in the Attenuated Total Reflectance (ATR, GladiATR^TM^, Pike technologies, USA) mode. Spectra were obtained at 4 cm^−1^ resolution co-adding 50 scans over a range of wavenumbers from 400 cm^−1^ to 4000 cm^−1^. Before analysis the samples were milled for 2 min at 30 rpm using mini-mill (Pulverisette 23, FRITSH, Germany).

### Preparation of osteogenic medium (OM)

8.8 g of DMEM powder and 3.3 g of NaHCO_3_ were dissolved in 890 ml of water. Obtained solution was supplemented with 100 ml of FBS superior serum, 10 ml of pen strep, 5 ml of 1 M β-glicerophosphate, 1 ml of 50 mg/ml L-ascorbic acid and 1 ml of 0.1 mM dexamethasone.

### Development and validation of UV/VIS spectroscopy method for the determination of SrRan content in OM

Analytical method for the SrRan content determination in OM using ultraviolet-visible spectroscopy (UV/VIS) has been developed and validated. Standard and sample solutions were measured against the OM in a dual beam UV-Visible spectrophotometer (VIS (UV/VIS spectrophotometer Evolution 300, Thermo Scientific, Waltham, MA) at 325 nm. Sample preparation for linearity, precision and accuracy tests included SrRan dissolution in OM for 80 min at 600 rpm.

The specificity of the analytical procedure was assessed by comparing the UV/VIS spectra of OM and SrRan standard solutions in OM.

The detection linearity of SrRan was verified by five-point calibration over the concentration range from 0.8–80 µg/ml. The limit of detection and limit of quantification of SrRan were calculated using results obtained within the linearity test according to the following equations:

2 $${\rm{LOD}}=3.3{\rm{\sigma }}/{\rm{B}},$$

3 $${\rm{LOQ}}=10{\rm{\sigma }}/{\rm{B}},$$

where: LOD – detection limit of the procedure, % of API test concentration; LOQ – quantitation limit of the procedure, % of API test concentration; σ – standard deviation of y-intercepts from regression line; B – slope of the trendline.

The repeatability of the procedure was assessed via analysis of 6 replicate SrRan samples with concentration of 10 µg/ml and results were expressed as a mean recovery rate. The repeatability test was performed three times, in two different days by two operators and the highest relative standard deviation (RSD) value was set as the overall repeatability of the procedure. The RSD between mean analyte content determined within three repeatability tests was considered as the intermediate precision.

The accuracy of the analytical procedure was assessed by the analysis of nine SrRan samples (three different concentrations (1 µg/ml, 10 µg/ml and 80 µg/ml) over the working concentration range). The accuracy of the procedure was expressed as the relative recovery rate, e.g. percent ratio between the determined content and the nominal content of analyte in the sample.

### Determination of SrRan release profiles *in vitro*

To evaluate *in vitro* SrRan release from the prepared microcapsules, 100 mg of three replicate samples from each microcapsule batch were immersed in 20 ml of OM and incubated at 37 °C ± 0.5 °C and 50 rpm (Environmental Shaker – incubator ES-20, Biosan, Riga, Latvia). 2 ml aliquots of the solution were taken directly from the vessels after 2 h, 4 h, 6 h, 8 h, 10 h, 24 h, then once every following day for a period of 7 days and finally at least once a week for a period of 120 days in case of s/o/w technique and 121 days in case of s/w_1_/o/w_2_ technique. The volume taken was replaced with 2 ml of fresh solution, keeping the total dissolution medium volume constant. SrRan content in dissolution medium was determined using UV/VIS at λ = 325 nm and expressed as the % of SrRan released from the total drug content in microcapsules.

### Microcapsule sample and extract preparation

Microcapsule suspensions for *in vitro* testing were prepared by suspending microcapsules at concentrations of 500 and 250 µg/ml^26^ in Dulbecco’s Modified Eagle Medium (DMEM high glucose, D6429, Sigma Aldrich) with 100 µg/mL streptomycin and 100 U/mL penicillin.

The extract of SrRan loaded microcapsules was prepared as follows: 20 mg of microcapsules were placed into a 15 ml polypropylene tube (Sarstedt), and suspended in 5 ml of DMEM culture medium with 100 µg/mL streptomycin and 100 U/mL penicillin. The DMEM culture medium with streptomycin/penicillin was used as a control. The tubes were then incubated at 37 °C in a humidified atmosphere of 5% CO2 for 24 h. Then, the medium was collected and filtered using sterile syringe filters (Filtropur S, 0.2 µm, Sarstedt).

Obtained extracts and microcapsule suspensions were used for *in vitro* viability assay.

### Evaluation of cell viability

The human osteosarcoma cell line MG-63 (ATCC® Number: CRL-1427™) was cultured with DMEM supplemented with 10% fetal bovine serum, 100 µg/mL streptomycin, and 100 U/mL penicillin in a CO_2_ incubator at 37 °C (in a humidified atmosphere of 5% CO2). After reaching 80% confluence, the cells were subcultured in 96-well plates at concentration of 3–10 × 10^4^ cells/ml (100 µl medium in each well). After overnight incubation at 37 °C, the culture medium was removed and replaced by 100 μl control medium; extract of blank and SrRan loaded microcapsules; or suspension of respective microcapsules at concentrations of 250 and 500 µg/ml. The viability of MG-63 cells was tested after 24, 48 and 72 h incubation with the extracts or after 72 h incubation with microcapsule suspensions and values are represented as the mean ± SD of at least 3 independent measurements in 6 parallels. MTT assay was performed according to ISO 10993-5:2009(E) standard^27^.

### Statistical evaluation

All results were expressed as the mean value ± standard deviation (SD) of at least three independent samples. The significance of the results was evaluated using unpaired Student’s t-test with the significance level set at p > 0.05. One-way and two-way analysis of variance (ANOVA) was performed to evaluate the differences between the results.

## Results and Discussion

### Development and validation of the analytical procedure for the UV/VIS determination of SrRan content in OM

A rapid and specific UV/VIS analytical method for the determination of SrRan content in OM has been developed and validated. The specificity of the analytical method was proven by comparing the UV/VIS spectra of osteogenic medium and SrRan standard solutions in OM.

The correlation coefficients of obtained calibration curves were evaluated and the absorbance of the SrRan in the range from 0.8–80 µg/ml was linear with a correlation coefficient R^2^ > 0.9999. The LOD and LOQ were calculated as 0.161 µg/ml and 0.488 µg/ml, respectively. Precision study of the developed method (Table 2) confirmed method reliability and the repeatability of the procedure was found to be 1.04%, while intermediate precision was set as 0.47%.

**Table 2:** Assessment of method precision.

	Mean SrRan recovery ± SD, %	RSD between 6 replicate samples, %
1^st^ Precision test	99.73	0.64
2^nd^ Precision test	99.18	0.69
3^rd^ Precision test	100.12	1.04

During the study it was found that accurate method is ensured and average recovery of SrRan within the range of 1–80 µg/ml is 100 ± 2.14% (Table 3).

**Table 3:** Assessment of method accuracy.

Concentration of SrRan in sample, µg/ml	Mean SrRan recovery ± SD, %
1	98.51 ± 0.70
10	100.66 ± 0.78
80	99.60 ± 1.00

### Preparation and characterization of SrRan loaded microcapsules using s/o/w technique

**Figure 1:**
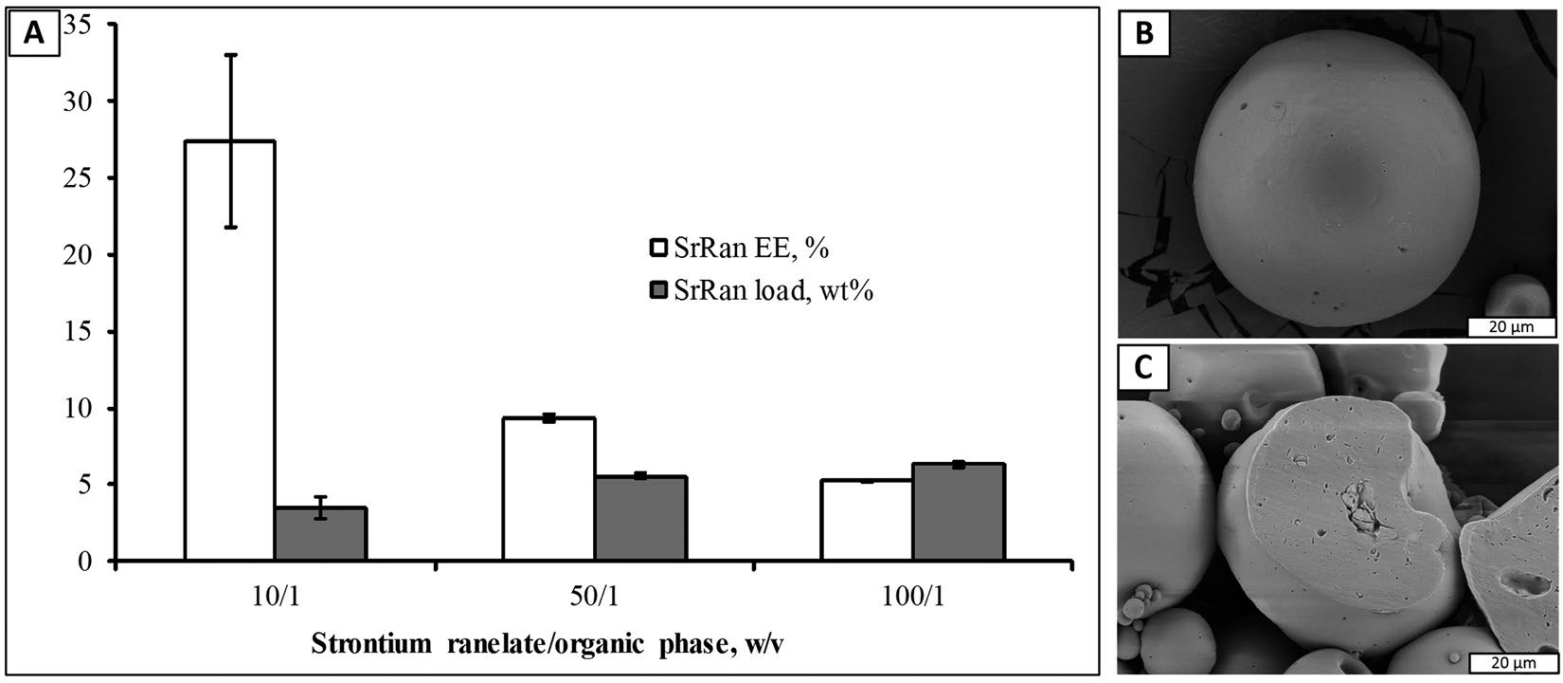
Characterization of SrRan loaded microcapsules prepared via s/o/w technique: **(A**) Impact of drug amount in the organic phase on the SrRan load and encapsulation efficiency; (**B**) SEM microphotographs of PLA/SrRan microcapsule surface (SrRan/organic phase ratio 10/1 (w/v)); (**C**) SEM microphotographs of PLA/SrRan microcapsule cross section (SrRan/organic phase ratio 10/1 (w/v)).

An attempt was made to microencapsulate slightly water soluble (solubility in water ~3 mg/ml) SrRan in PLA matrix using solid/in oil/in water (s/o/w) microencapsulation technique. Impact of SrRan amount in the organic phase on the total drug load and encapsulation efficiency was evaluated (Fig. 1A).

It was expected that the increase of active ingredient content in the organic phase will significantly increase the total drug load, while only a small decrease in encapsulation efficiency was foreseen. However, increase of SrRan content in the organic phase by 10 times, increased the total drug content in microcapsules only from 3.47 ± 0.69 wt% (SrRan/organic phase ratio 10/1) up to 6.31 ± 0.14 wt% (SrRan/organic phase ratio 100/1), while sharp decrease in the encapsulation efficiency from 27.39 ± 5.64 wt% down to 5.24 ± 0.02 wt% (SrRan/organic phase ratio 50/1) was observed.

SEM investigations revealed that SrRan loaded microcapsules have a spherical shape and smooth surface (Fig. 1B). Some crystals of the active substance were also observed on the surface of the microcapsules. The cross-sections of the obtained drug-loaded microcapsules showed that they are comprised of discrete active substance loaded domains from 2 to 15 µm in diameter (Fig. 1C). Particle size and particle size distribution of the obtained microcapsules was not significantly affected by the SrRan/organic phase ratio changes and was in the range from 19–84 μm (Table 4).

**Table 4:** Particle size distribution of SrRan loaded PLA microcapsules prepared via s/o/w technique.

SrRan/organic phase ratio, (w/v)	Particle size ± SD, μm		
	d_10_	d_50_	d_90_
10/1	19 ± 1	39 ± 1	77 ± 6
50/1	19 ± 1	42 ± 3	84 ± 14
100/1	20 ± 1	42 ± 6	84 ± 3

**Figure 2:**
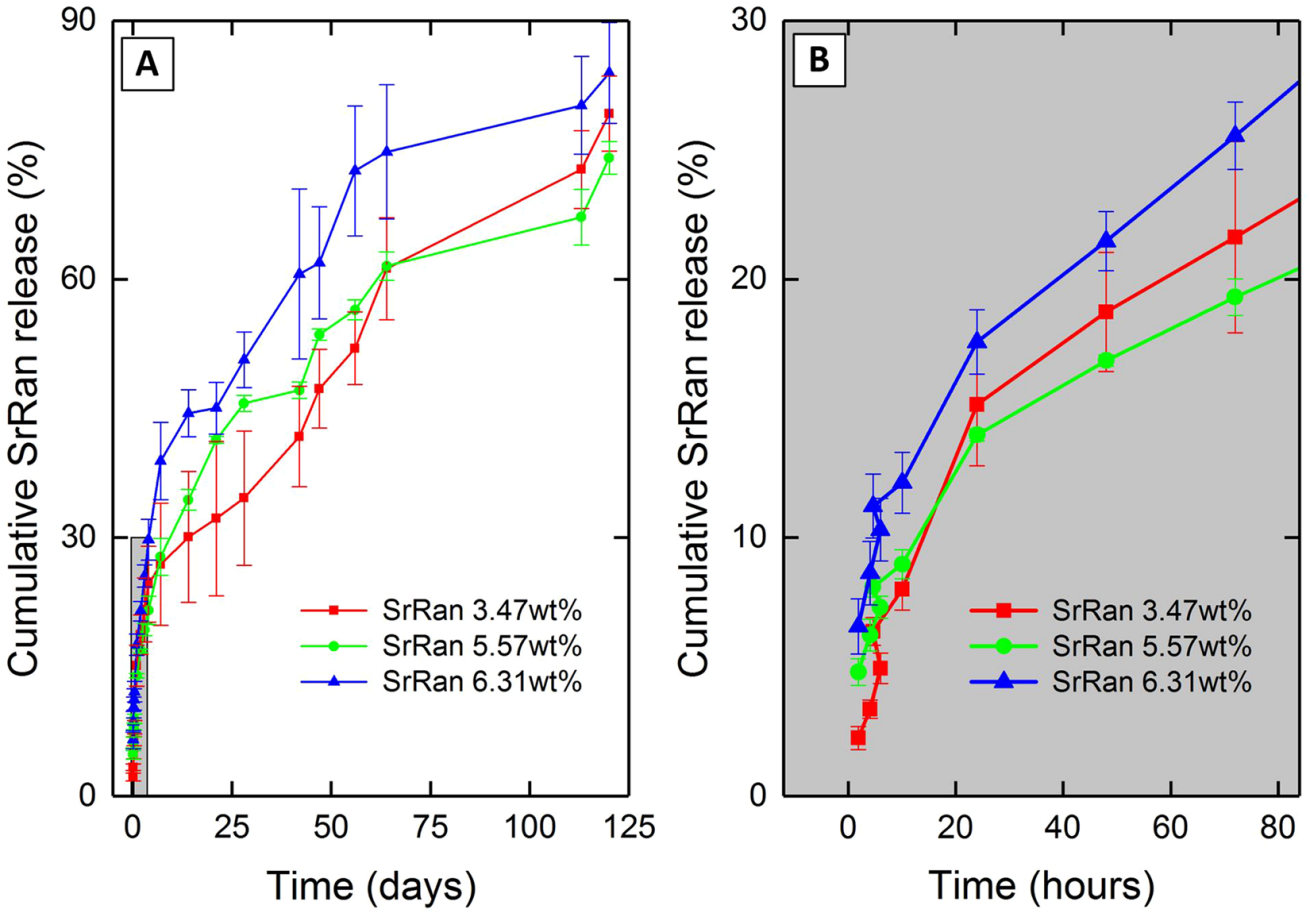
Effect of SrRan/organic phase ratio on: (**A**) strontium ranelate release from microcapsules; (**B**) strontium ranelate initial burst release within the first 24 h from microcapsules.

*In vitro* SrRan release in osteogenic medium from the microcapsules prepared with different SrRan/organic phase ratio was studied for 120 days (Fig. 2).

It was found that within the first 24 h (Fig. 2B) 15% of active substance was released and significant effect of SrRan/organic phase ratio on the drug initial burst release was not observed. The substantial burst release could be attributed to the dissolution of active substance crystals found on and close to the surface of the microcapsules. During the first two weeks no significant effect of SrRan/organic phase ratio on the release kinetics of the drug was observed. Significant differences (p < 0.05) for SrRan release kinetics appears starting from day 14 up to day 56 and were observed only comparing the microcapsules with the lowest and the highest total drug load (Fig. 2A).

### SrRan loaded microcapsules prepared via s/w_1_/o/w_2_ technique

In order to increase the total drug load in microparticles, s/w_1_/o/w_2_ technique was applied for the SrRan microencapsulation in PLA matrix. For the preparation of s/w_1_ phase ultrasound homogenization was used, thus resulting in facilitated SrRan solubility in water (from 3 mg/ml to 10 mg/ml) and significantly decreased (for more than 4 times) mean particles size of the active substance. Characterization of microcapsules prepared via s/w_1_/o/w_2_ technique is given in Table 5.

**Table 5:** Characterization of microcapsules prepared via s/w_1_/o/w_2_ technique.

SrRan/organic phase ratio, (w/v)	SrRan load ± SD, wt%	SrRan EE ± SD, %	Particle size ± SD, μm		
			d_10_	d_50_	d_90_
10/1	3.57 ± 0.28 (SrRan 4 wt%)	37.53 ± 3.70	18 ± 1	40 ± 2	74 ± 7
50/1	16.39 ± 0.50 (SrRan 16 wt%)	40.20 ± 4.43	19 ± 1	42 ± 2	71 ± 1
100/1	24.39 ± 0.91 (SrRan 24 wt%)	28.36 ± 2.27	19 ± 1	42 ± 1	69 ± 4

**Figure 3:**
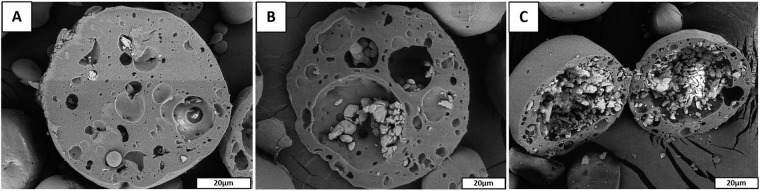
SEM microphotographs of microcapsules prepared using ultrasound homogenization of w_1_ phase: (**A**) SrRan/o phase ratio 10/1(w/v), total drug load 4 wt%; (**B**) SrRan/o phase ratio 50/1(w/v), total drug load 16 wt%; (**C**) SrRan/o phase ratio 100/1(w/v), total drug load 24 wt%.

Increasing SrRan/organic phase w/v ratio from 10/1 to 100/1 it was possible to prepare the microcapsules with strontium ranelate load from 3.57 ± 0.28 wt% up to 24.39 ± 0.91 wt% and drug encapsulation efficiency from 37.5 ± 3.7% to 28.4 ± 2.3%, respectively. It was found that addition of the water phase did not influence the particle size of prepared microcapsules. SEM investigation of obtained microcapsules revealed that upon increasing SrRan/organic phase ratio, the size of drug loaded polymer domains increased (Fig. 3).

**Figure 4:**
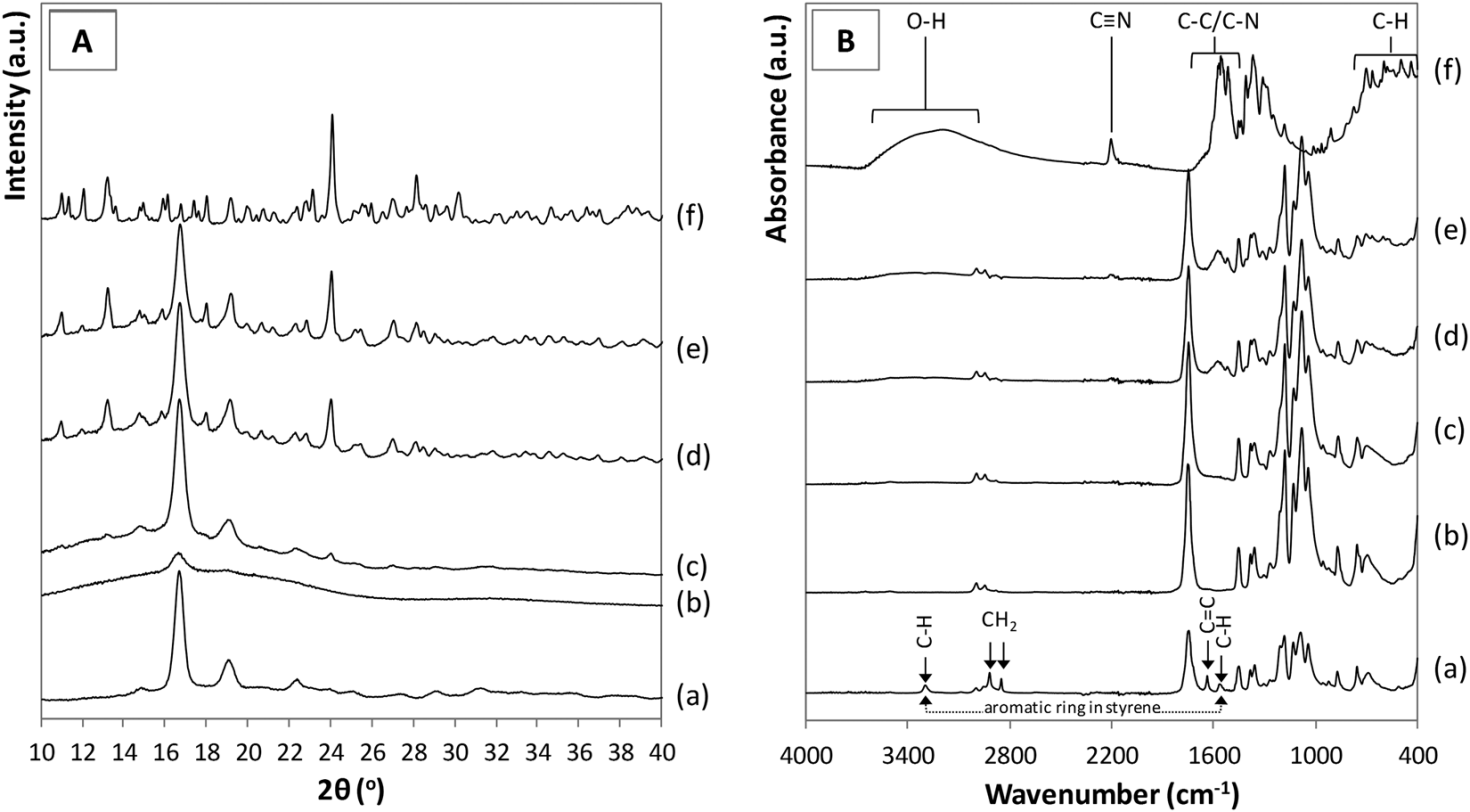
(**A**) XRD patterns and (**B**) FT-IR spectra of (a) commercial PLA, (b) PLA film, (c) SrRan 4 wt%, (d) SrRan 16 wt%, (e) SrRan 24 wt% and (f) commercial SrRan.

Interaction between the polymer and drug, as well as influence of the microcapsule preparation process on the raw materials was studied by XRD and FT-IR (Fig. 4A,B, respectively).

The presence of PLA in the microcapsules was evidenced by relatively well resolved XRD peaks at 16.7° and 18.9^o^ 2θ and significant amorphous background hump (Fig. 4A(c,d)), which is indicative for the semi-crystalline structure of the PLA. Whereas, crystalline SrRan exhibited well resolved XRD peak set as described in^28^ (Fig. 4A (f)). As expected, increase in intensity of the SrRan XRD peaks with increasing SrRan content was observed (Fig. 4A(c,d)). FT-IR results correlate with XRD and confirm the presence of main two components of the microcapsules – PLA and SrRan. The region of spectra of commercial PLA (Fig. 4B(a)), PLA film (obtained from solution casting) spectra (Fig. 4B(b)), and the microcapsules (Fig. 4B(c,d)) between 870 and 1450 cm^−1^ contains number of modes which are characteristic to neat PLA and are attributable to C-O-C, C-C vibrations^29^. In addition, absorbance bands indicated with arrows in FT-IR spectra of the commercial PLA (Fig. 4B(a)) implies a presence of acrylonitrile butadiene styrene (ABS) residues from the production of PLA, where it is commonly used as a plasticizer^30^. Nevertheless, these absorbance bands were not detected in spectra of the microcapsules, as during the preparation of microcapsules, PLA undergoes the recrystallization. Increase of SrRan content in the microcapsules was confirmed by the corresponding intensity changes of the absorbance bands assigned to SrRan (Fig. 4B(f)). There were no significant differences observed between polymer/drug interactions depending on the microcapsule preparation technique.

**Figure 5:**
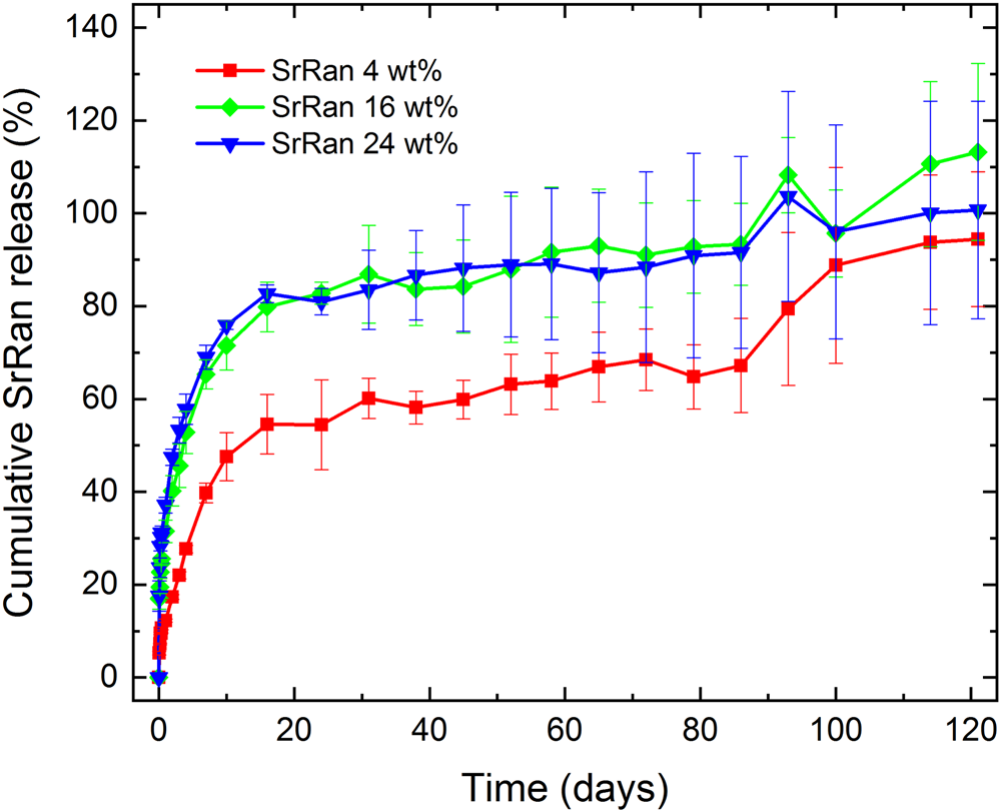
SrRan release from microcapsules prepared via s/w_1_/o/w_2_ technique

*In vitro* strontium ranelate release in osteogenic medium from the microcapsules prepared via s/w_1_/o/w_2_ technique was studied for 121 days (Fig. 5).

**Figure 6:**
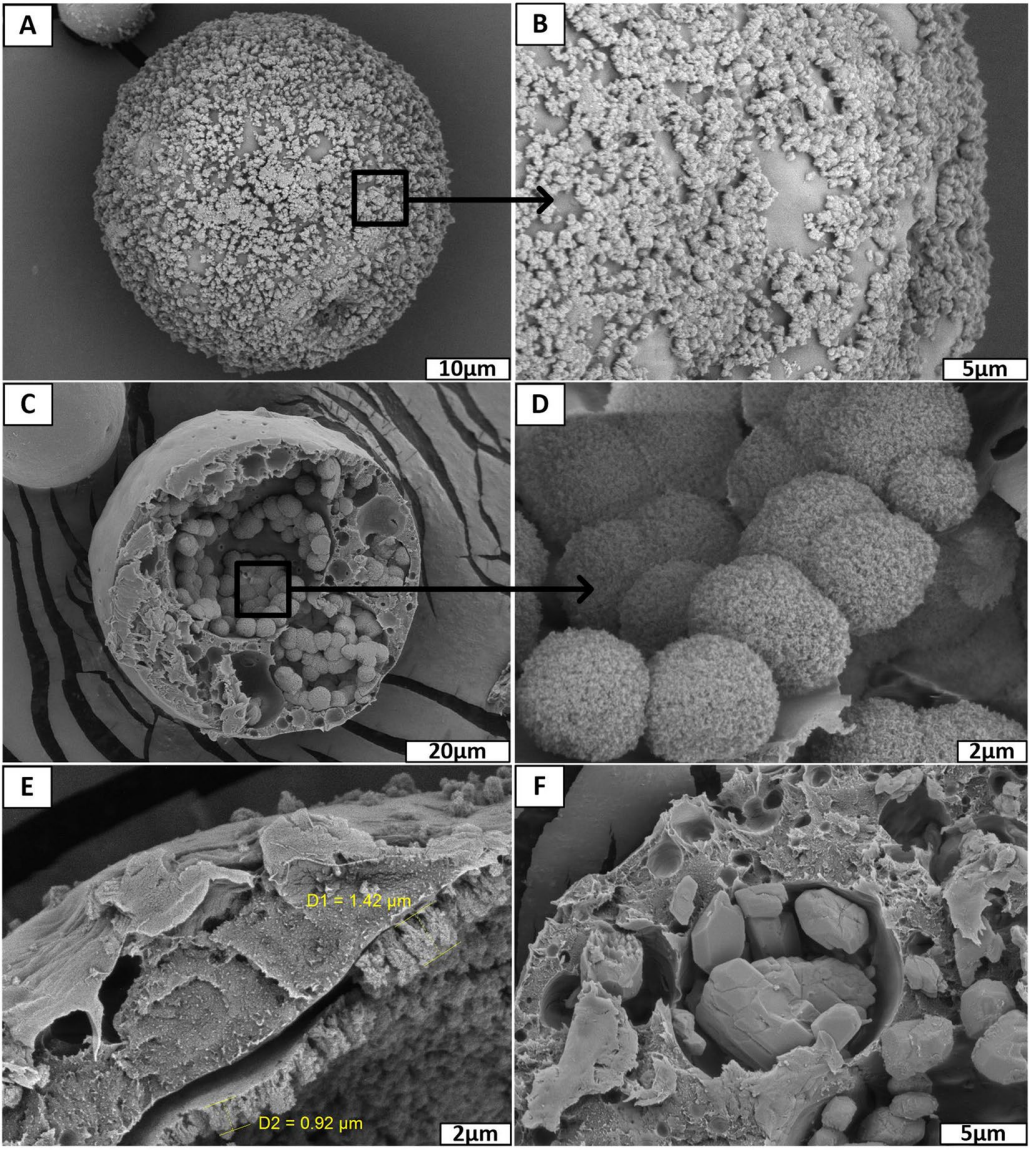
SrRan loaded PLA microcapsules prepared via s/w_1_/o/w_2_ technique after 121 days in osteogenic medium: (**A**,**B**) surface evaluation; (**C**–**F**) inner structure evaluation.

It was found that the drug release rate depends on the SrRan content in the microcapsules. In the first 16 days microcapsules containing 24 wt% and 4 wt% of SrRan showed 82.67 ± 1.92% and 54.56 ± 1.92% release of active ingredient in the OM (Fig. 5), respectively. It was observed that starting from day 30 standard deviations between replicate samples significantly increased and this tendency was more pronounced for those microcapsules with higher drug loads. At day 100, differences among replicate samples became more than ±20%. Although results showed that active substance from the microcapsules have been completely released already upon 100 days, slight increase in the UV/VIS absorption intensity at 325 nm was still observed in day 114 and 121. To identify these findings, SEM investigations of the morphology and inner structure of microcapsules after 121 days in OM was performed (Fig. 6).

Obtained results showed, that drug particles can be still found in some closed pores of microcapsules (Fig. 6F), indicating that the release of the SrRan have not been completed within 121 days, contrary to the results obtained from UV/VIS spectroscopy. It was suggested that despite the application of NaN_3_ as a preservative, some changes have occurred in OM that led to the increased background signal of the dissolution medium at 325 nm, resulting in deceptive increase of UV/VIS signal, deviations among replicate samples and, thus, calculated amount of SrRan content in the dissolution medium.

**Figure 7:**
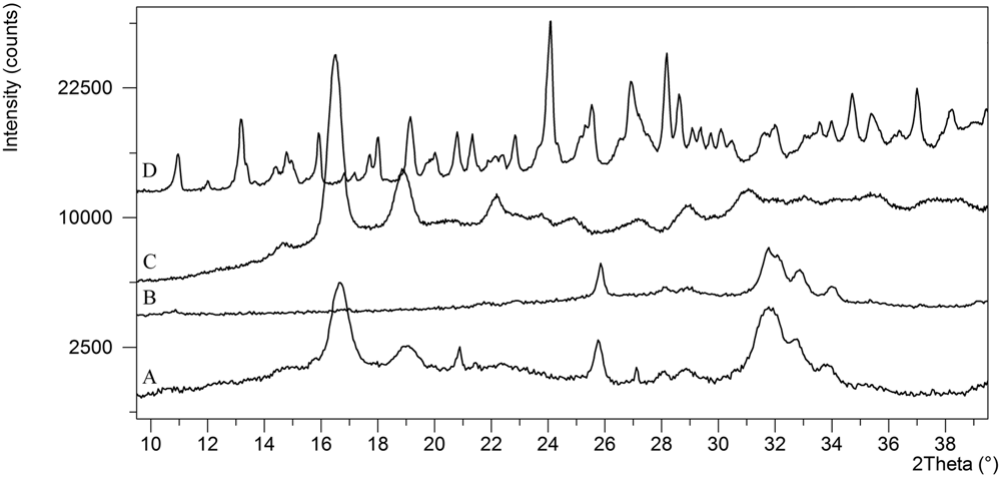
XRD analysis of: (**A**) SrRan loaded microcapsules after 121 days in OM, (**B**) nanocrystalline precipitated hydroxyapatite; (**C**) commercial PLA; (**D**) commercial SrRan.

SEM investigation of SrRan loaded PLA microcapsules incubated for 121 days in OM, revealed the formation of biomimetic calcium phosphate precipitates on the surface of microcapsules (see Fig. 6A and B). Moreover, significant amount of precipitated calcium phosphate with characteristic spherical morphology for apatites was also found in the inner voids of microcapsules (see Fig. 6C,D) as well as precipitated film type layer of calcium phosphate (1 to 1.5 µm thin) on the pore walls was observed. To identify the chemical composition of observed precipitates, XRD analysis of microcapsules incubated for 121 days in OM was performed (see Fig. 7).

Biomimetic calcium phosphate in the form of hydroxyapatite was identified. Obtained XRD pattern corresponds to the hydroxyapatite found in the ICDD database #01-072-1243. It was suggested, that the formation of calcium phosphate (most probably carbonated calcium deficient hydroxyapatite^31^) in the microcapsules was ensured by the diffusion of OM through the open channels in the pore walls.

**Figure 8:**
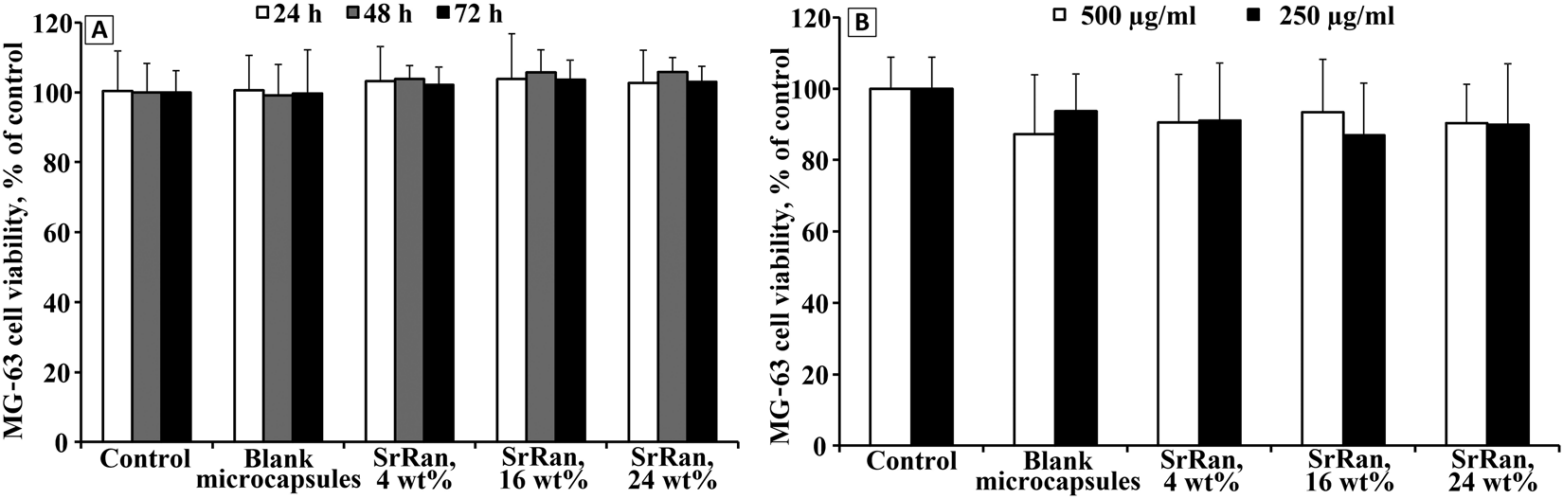
Effects of blank and SrRan loaded microcapsule extracts (**A**) and microcapsule suspensions (**B**) on cell viability measured by MTT assay.

Evaluation of *in vitro* cell viability on human osteosarcoma cell line MG-63 showed that there is no evidence of cytotoxic effect of microcapsule extracts, with both blank and SrRan loaded microcapsules (see Fig. 8A). The amount of encapsulated SrRan up to 24 wt% does not affect the cell viability. Moreover, the cell viability evaluation of microcapsule suspensions approved that even at the concentration of 500 µg/mL, no cytotoxic effects were observed after 72 h of incubation for both blank and SrRan loaded microcapsules (see Fig. 8B).

## Conclusions

It was established that the s/w_1_/o/w_2_ method is applicable for the preparation of microencapsulated sustainable release SrRan delivery systems containing up to 24 wt% of active substance. To increase the drug load in the microcapsules and encapsulation efficiency of active ingredient ultrasound should be applied for the s/w_1_ phase homogenization. Accordingly, ultrasonication significantly decreased the active substances mean particles size and facilitated the drug solubility in the water. Prepared delivery systems can ensure sustained SrRan release in osteogenic medium for more than 121 days.

Precipitation of biomimetic calcium phosphate on the surface and in the pores of prepared delivery systems was observed after microcapsule exposure to OM for 121 days. In order to reveal the biomimetic calcium phosphate precipitation initiation mechanisms and kinetics, further detailed investigations are required.

## Data Availability

The datasets generated during and/or analyzed during the current study are available from the corresponding author on reasonable request.
